# Examination of novel *Aureobasidium pullulans* isolates dominating apple microflora and assessing their potential for apple juice spoilage

**DOI:** 10.1007/s11274-018-2497-5

**Published:** 2018-07-11

**Authors:** Łukasz Wajda, Magdalena Wyderka, Zuzanna Polak, Aleksandra Duda-Chodak, Małgorzata Makarewicz

**Affiliations:** 1 Malopolska Centre of Biotechnology, Jagiellonian University, Gronostajowa 7A str, 30-387 Krakow, Poland; 2 Department of Fermentation Technology and Technical Microbiology, Faculty of Food Technology, University of Agriculture in Krakow, ul. Balicka 122, 30-149 Krakow, Poland

**Keywords:** *Aureobasidium pullulans*, Fungi, Apple, Juice

## Abstract

There is very little up to date information regarding apple microflora so in the current study we decided to address that issue and assess whether dominant fungi which reside in fruit might spoil apple juice. Microorganisms were isolated from apples of Koksa Górska harvested in the middle of October in 2016 and 2017. Identification of isolates was based on the sequencing of ribosomal DNA. Some isolates were characteristic for a particular year but in both years apple microflora was dominated by *Aureobasidium pullulans*. Based on phylogenetic analysis it was stated that only one isolate (LW81) was closely related to strains which are already described in available databases. All other isolates collected in the current study differed significantly from sequences stored in databases, tending to form a common cluster. It was possible to predict secondary structure of ITS2 region only for the isolate LW81, while we managed to establish the length and location of 5.8S gene in Rfam database for all sequences. *A. pullulans* is known exopolysaccharide producer so obtained microorganisms were tested for their ability to synthesise those substances on Czapek-Dox agar. The strain which proved to be the most significant exopolysaccharide producer (isolate LW14) was inoculated in the sterilised apple juice at three different initial cell number (100, 1000 and 10,000 cfu/ml) and subjected to pasteurisation. In all cases pasteurisation eliminated fungal growth effectively, therefore *A. pullulans* strains should not pose any risk to the quality of pasteurised apple juices.

## Introduction

Knowledge about microbial species residing in raw materials, e.g. fruits or vegetables, is essential for predicting issues related to post-harvest losses or spoilage of fruit products, i.e. juices, jam, marmalades etc. Moreover, when food manufacturers are aware of microorganisms which might deteriorate the quality of finished goods, they are able to select adequate food processing methods to provide the highest quality of their products, e.g. by adjusting parameters of thermal processing, introducing certain chemicals for washing and disinfection which are effective against fungi etc. Since apple is one of the most popular fruit in Poland, we decided to address identification of fungi residing in apple which might deteriorate the quality of apple-based products. Literature available on that subject is very scarce, and mostly, outdated. Majority of the research was carried out before molecular methods became commonly available (Williams 1955; Pennycook and Newhook 1981; Bernier et al. 1996). Moreover, most studies focused on changes of apple microflora during cold storage of harvested fruits (Mirzwa-Mróz et al. 2012; Alwakeel 2013) or on mould known as common apple pathogens (Grantina-Ievina 2015). It has been demonstrated that various fungal species might survive pasteurisation, i.e. *Zygosaccharomyces bailli, Candida krusei, Saccharomyces bisporus, Schizosaccharomyces pombe, Pichia membranifaciens, Byssochlamys fulva, B. nivea, Neosartorya fischeri*, and *Talaromyces* (Aneja et al. 2014) but there are also others which might cause economical losses in the manufacturing of juices or soft drinks. Their presence in beverages could be related to the quality of raw materials and poor hygiene practices (Wareing and Davenport 2007). Most of species isolated from juices and production plants are non-pathogenic, however, those microorganisms synthetase compounds that are unwanted in food products.

Since most of the data regarding apple microflora is outdated, we decided to identify fungal species residing on apples in two subsequent years. In our unpublished study, we demonstrated that apple microflora is dominated by *Aureobasidium pullulans* strains. Microorganisms belonging to that species used to be divided into four varieties: *pullulans, melanogenum, subglaciale* and *namibiae* (Zalar et al. 2008), however, after in-depth genetical analysis, they were redefined as separate species (Gostinčar et al. 2014). In the current research, we decided to examine *A. pullulans* isolates obtained from apple cultivar (Koksa Górska) which is used by local producers of apple juices (Łososina, Poland). The study was carried out in two subsequent years (2016 and 2017). We subjected the structure of obtained sequences to detailed analysis using software available for that purpose. Then we assessed the possibility of juice spoilage by the most potent exopolysaccharide producers.

## Materials and methods

Microbiological media were purchased from BIOCORP (Gliwice, Poland) and chloramphenicol was obtained from A&A Biotechnology (Gdańsk, Poland). Genomic Bead-Beat Micro AX Gravity kit (DNA isolation) and Clean-up AX (purification of PCR products) were purchased from A&A Biotechnology (Gdańsk, Poland). The following chemicals were used for the PCR: One *Taq* DNA Polymerase with reaction buffer (Lab-Jot, Warszawa, Poland), dNTP’s mix (Symbios, Straszyn, Poland), sterile water (Aqua pro injection, Polpharma SA Pharmaceutical Works, Gdansk, Poland), and primers (Genomed S.A., Warszawa, Poland) ITS1 (5′-TCCGTAGGTGAACCTGCG-3′) and ITS4 (5′-TCCTCCGCTTATTGATATGC-3′); or M13 (5′-GTT TTC CCA GTC ACG AC-3′). Reagents for electrophoresis were Agarose Biotechnological Grade (Bioshop Canada Inc., Burlington, Ontario, Canada), SimlySafe dye (EURx Ltd., Gdańsk, Poland), DNA ladder for RAPD-PCR (100 bp ladder, Amresco LLC, Canada) and DNA ladder for PCR with ITS primers (100 bp DNA Ladder RTU, GeneDireX Inc., Las Vegas City, Nevada, USA). Apple juice concentrate (Ambar-Aldo, Brzezie, Poland) was used for the preparation of sterilised apple juice.

### Isolation of fungal strains

Fruit of Koksa Górska (Łososina, Poland) was harvested in the middle of October 2016 and October 2017. This cultivar was selected because it was popular among local manufacturers of natural apple juices (personal hearing). In both years fruits were collected directly from trees and immediately delivered to the laboratory for analysis. Homogenised fruit (5 min, Ultra Turrax T-25-basic homogeniser, IKA-Werke GmbH & Co. KG, Staufen, Germany) was used for the preparation of serial dilutions with peptone–saline water. Peptone–saline water was a solution containing 0.1% of bacteriological peptone and 0.85% of sodium chloride. For the pour plate method, WL (Wallerstein Laboratory) nutrient agar supplemented with chloramphenicol (100 mg/l) was used. After incubation (72 h/28 °C), colonies which were most abundant or the most characteristic for each cultivar were selected and streak plated on fresh WL nutrient agar. Morphology of each colony was annotated and microscopic observations were carried out for each of them. In 2016, 13 isolates were obtained, while in 2017 it was 20 isolates. Strains were stored at 4 °C and were transferred on fresh agar slants every 3 weeks.

### DNA isolation and PCR

Before DNA isolation, each fungal isolate was incubated at 28 °C for 24 h (72 h for putative mould isolates) in Sabouraud dextrose broth (10 ml) supplemented with chloramphenicol (100 mg/l) in 100 ml Erlenmayer flasks on the rotary shaker (Orbit 1000, Labnet International Inc., Edison, NJ, USA) at 120 rpm. Then cell suspension was centrifuged (2750×*g*/15 min, MPW-350R, MPW Med. Instruments, Warszawa, Poland). After washing with 5 ml of sterile deionised water (2750×*g*/15 min) obtained biomass was re-suspended in 1 ml of sterile deionised water. The final suspension was centrifuged at 14,000×*g*/1 min (MPW-65R, MPW Med. Instruments, Warszawa, Poland) and genomic DNA was obtained using Bead-Beat Micro AX Gravity assay kit according to the manufacturer’s instructions. Mould biomass was subjected to three cycles of freezing (− 86 °C/24 h)—thawing (50 °C/30 min) before isolation. Obtained DNA was kept at − 20 °C until further analysis. The reaction mixture contained 26.75 µl of sterile water, 10 µl of 5× One *Taq* standard reaction buffer, 0.25 µl of One *Taq* DNA Polymerase (5000 U/ml), 1 µl of dNTP’s, 1 µl of forward ITS1 primer, 1 µl of reverse ITS4 primer and 10 µl of the DNA template. The reaction was carried out in a Multigene Mini thermocycler (Labnet International, Edison, NJ, USA) as described by Fujita et al. (2001). The electrophoresis of agarose gels (1.7% w/v, 5 µl of Simply Safe dye/100 ml) was carried out for 60 min/100 V against a 100 bp DNA RTU ladder. The reaction mixture for RAPD-PCR (50 µl) was the same as for the reaction with ITS primers but it contained 1 µl of M13 primer. RAPD-PCR and gel electrophoresis afterwards were carried out as described previously (Andrighetto et al. 2000). In some cases, it was necessary to prepare a ten-fold dilution of the DNA template for PCR and RAPD-PCR to dilute natural inhibitors of PCR.

### DNA sequencing and data interpretation

Clean-up AX kit was used for the purification of the DNA obtained after amplification and diluted with nuclease free water to 50 ng/µl. 5 µl of the DNA solution was combined with 5 µl of ITS1 or ITS4 primer (5 pmol/µl). DNA sequencing was carried out by Macrogen Europe (Amsterdam, Netherlands) using the Sanger deoxynucleotide method. Identification of fungal strains was based on the amplification of partial 18S rDNA, ITS1 region, 5.8S rDNA, ITS2 region and partial 28S rDNA. Sequences were edited in BioEdit, version 7.2, 2005 (Hall 1999) and ambiguous fragments at the beginning and the end of obtained sequences were trimmed. Obtained results were analysed in a BLAST search engine (https://blast.ncbi.nlm.nih.gov/Blast.cgi) and those for which identity was at least 98% conclusive were submitted to the NCBI, accession numbers were assigned to each strain.

### Building phylogenetic tree

The analysis was performed on the phylogeny.fr platform (Dereeper et al. 2008). Sequences were aligned with MUSCLE (v3.7) configured for highest accuracy (Edgar 2004). After alignment, ambiguous regions (i.e. containing gaps and/or poorly aligned) were removed with Gblocks (v0.91b) (Castresana 2018) using the following parameters: minimum length of a block after gap cleaning-10, no gap positions were allowed in the final alignment, all segments with contiguous nonconserved positions bigger than eight were rejected, minimum number of sequences for a flank position was 85%. The phylogenetic tree was reconstructed using the maximum likelihood method implemented in the PhyML program (v3.0) (Guindon et al. 2010). The HKY85 substitution model was selected assuming an estimated proportion of invariant sites and four gamma-distributed rate categories to account for rate heterogeneity across sites. The gamma shape parameter was estimated directly from the data. Reliability for internal branch was assessed using the aLRT test (SH-Like) (Street and Kingdom 2006). Graphical representation and edition of the phylogenetic tree were performed with TreeDyn (v198.3) (Chevenet et al. 2006). All branches with support values < 70% were dropped. For phylogenetic analysis *Kabatiella microsticta* strain CBS 342.66 (accession number EU167608.1) was selected as an outgroup since it was closely related to most tested microorganisms.

### Predicting sequence structures

The sequence of ITS2 region and its structure was modelled with ITS2 Database (Koetschan et al. 2009; Merget et al. 2012). The sequence of 5.8S region was modelled with Rfam (Nawrocki et al. 2015; Kalvari et al. 2018) on http://rfam.xfam.org after converting DNA to RNA.

### Screening for exopolysaccharide producer and its resistance to pasteurisation

All *A. pullulans* strains obtained in the current study were streaked on Czapek-Dox agar supplemented with chloramphenicol (100 mg/l). This medium contains sucrose which has been demonstrated to be the most optimum carbon source for producing exopolysaccharides by *A. pullulans* (Cheng et al. 2011). Plates were incubated for 5 days at 28 °C and checked for the formation of slimy colonies. Isolates which produced the most significant quantities of exopolysaccharides on plates were examined against their ability for producing those substances in liquid medium as described by Yoon et al. (2012). Tests were carried out at three different initial cell number [cfu/ml]: 1000, 10,000 and 100,000. We verified that 1,000,000 cfu/ml of tested strain equals 1.25 McFarland measured with DEN-1B McFarland Densitometer (Biosan, Riga, Latvia).

Apple juice was prepared with apple juice concentrate and its final parameters were as follows: total acidity expressed as malic acid 10 g/l, pH 3.5 and extract 10 °Bx. After sterilisation, apple juice was cooled down and stored at 4 °C for further experiments but no longer than 24 h. The strain of *A. pullulans* that produced the most exopolysaccharides on Czapek-Dox medium was inoculated in sterilised apple juice (10 ml) and incubated on the Orbit 1000 rotary shaker 24 h before the experiment. Then obtained fungal suspension was diluted with sterilised apple juice to 10,000 cfu/ml (suspension 1); 100,000 cfu/ml (suspension 2) and 1,000,000 cfu/ml (suspension 3). The rest of sterilised apple juice was divided into 99 ml aliquots to 100 ml capped sterile bottles. Each fungal suspension (1 ml) was added to five bottles with sterilised juice so final cell number was 100, 1000, and 10,000 cfu/ml. All experimental variants were kept in boiling water bath for 5 min immediately after inoculation, which was sufficient time for reaching 92 °C for 5 s. All bottles were transferred into the ice water bath. Cold juice was passed through the membrane filter (porosity 0.2 µm, cellulose nitrate filter, Sartorius Stedim Poland, Kostrzyn Wielkopolski, Poland) and filters were transferred on plates with WL nutrient agar supplemented with chloramphenicol (100 mg/l). All plates were incubated at 28 °C for 72 h, examined for colony growth and kept in the incubator for another 7 days.

## Results

### Identification of fungal isolates

The number of total fungi determined in fruit in 2017 was higher (1.37 ± 0.03 × 10^4^ cfu/g) than in 2016 (3.95 ± 0.55 × 10^3^ cfu/g). On the other hand, the number of isolates obtained in 2016 was much lower than in 2017 because isolates from 2016 were more difficult to maintain on agar slants. Microscopic observations were not useful for the initial identification of yeast, however, in the case of *A. pullulans* isolates (Table 1), the presence of characteristic fusiform cells was noted (Zalar et al. 2008). *A. pullulans* dominated apple microflora in both years but obtained isolates demonstrated great variability in colony appearance, considering colour and texture (Table 1). We identified microorganisms characteristic for each year of study, e.g. *P. membranifaciens* and *Candida railenensis* for 2016 or *Rhodotorula mucilaginosa* for 2017 (Table 1). Moreover, *Fusarium* and *Cladosporium* strains were found only among isolates obtained in 2017. Observation of microscope slides with mould isolates enabled identification of the *Alternaria* strains collected in 2016 to the genus level due to the presence of characteristic 8-transverse septate spores in (Table 1) but it was not helpful for identifying isolates LW78, LW23 and LW79.

**Table 1:** Description and identification of fungal strains from Koksa Górska cultivar

Year	Microorganism name	Isolate no.	Accession no.	Colony appearance/microscopic observations
2016	*Candida railenensis*	LW24	MG890257	Dark-blue and smooth colonies; smooth colony margins; no red pigment released to the growth medium/oval and cylindrical cells; no pseudohyphae or hyphae
	*Aureobasidium pullulans*	LW25	MG890258	Yellow-brown colonies; eroded, yellow colony margins; no red pigment released to the growth medium; butyrous texture/oval and cylindrical cells; no pseudohyphae or hyphae
	*Pichia membranifaciens*	LW26	MG890259	Off-white colonies with green colony margins; smooth colonies; eroded colony margins; no red pigment released to the growth medium/oval and cylindrical cells; no pseudohyphae or hyphae
	*Aureobasidium pullulans*	LW12	MG561921	Blue colonies; smooth colony margins; no red pigment released to the growth medium; butyrous texture/oval, round, fusiform and cylindrical cells; no pseudohyphae or hyphae
	*Aureobasidium pullulans*	LW10	MG561919	Dirty orange colonies; smooth colony margins; no red pigment released to the growth medium; butyrous texture/oval and cylindrical cells; no pseudohyphae or hyphae
	*Metschnikowia pulcherrima*	LW8	MG561918	Dark green colonies; smooth colony margins; red pigment released to the growth medium; butyrous texture/very small round and oval cells; no pseudohyphae or hyphae
	*Aureobasidium pullulans*	LW80	MG561917	Beige colonies with blue margins; eroded colony margins; no red pigment released to the growth medium; butyrous texture/oval, round and fusiform cells; no pseudohyphae or hyphae
	*Alternaria alternata*	LW76	MH095980	Black suede flat colonies/no septate hyphae; 8-transvere septate spores
	*Alternaria* sp.	LW77	MH095981	Black suede flat colonies/no septate hyphae; 8-transvere septate spores
2017	*Aureobasidium pullulans*	LW81	MG654656	Beige colonies with blue margins; eroded colony margins; no red pigment released to the growth medium; butyrous texture/oval, round and fusiform cells; no pseudohyphae or hyphae
	*Aureobasidium pullulans*	LW13	MG669474	Beige colonies; darker and eroded colony margins; no red pigment released to the growth medium; butyrous texture/oval and fusiform cells; no pseudohyphae or hyphae
	*Aureobasidium pullulans*	LW14	MG669476	Light-brown colonies; eroded colony margins; no red pigment released to the growth medium; butyrous texture/oval, round and fusiform cells; no pseudohyphae or hyphae
	*Rhodotorula mucilaginosa*	LW15	MG669558	Pink colonies; smooth colony margins; no red pigment released to the growth medium; slimy texture/oval and round cells; no pseudohyphae or hyphae
	*Aureobasidium pullulans*	LW16	MG669580	Dark-green colony centres; off-white, eroded colony margins; no red pigment released to the growth medium; consistent texture/oval and round cells; no pseudohyphae or hyphae
	*Aureobasidium pullulans*	LW17	MG669639	Blue colonies; smooth colony margins; no red pigment released to the growth medium; butyrous texture/oval, round, fusiform and cylindrical cells; no pseudohyphae or hyphae
	*Metschnikowia* sp.	LW18	MG669655	Light-green colonies; smooth colony margins; red pigment released to the growth medium; butyrous texture/very small round and oval cells; no pseudohyphae or hyphae
	*Aureobasidium pullulans*	LW19	MG669656	Yellow-brown colonies; smooth colony margins; no red pigment released to the growth medium; butyrous texture/oval, fusiform and cylindrical cells; no pseudohyphae or hyphae
	*Aureobasidium pullulans*	LW20	MG669657	Blue colonies; smooth colony margins; no red pigment released to the growth medium; butyrous texture/oval, round, fusiform and cylindrical cells; no pseudohyphae or hyphae
	*Aureobasidium pullulans*	LW21	MG670093	Yellow-brown colonies; smooth colony margins; no red pigment released to the growth medium; butyrous texture/oval, fusiform and cylindrical cells; no pseudohyphae or hyphae
	*Aureobasidium pullulans*	LW22	MG670094	Blue colonies; smooth colony margins; no red pigment released to the growth medium; butyrous texture/oval, round, fusiform and cylindrical cells; no pseudohyphae or hyphae
	*Fusarium* sp.	LW78	MH095979	Off-white, fluffy colonies; beige colony margins/septate hyphae; fusiform septate conidia
	*Fusarium* sp.	LW23	MG685829	Off-white, fluffy colonies; yellow-brown colony base; beige colony margins/septate hyphae; fusiform septate conidia
	*Cladosporium* sp.	LW79	MH095978	Dark-brown, suede colonies/septate hyphae; shield-shaped and fusiform conidia in chains

Band sizes obtained after PCR with ITS1 and ITS4 primers for isolates collected in 2016 differed between each other, while for some strains from 2017, band sizes were identical even if isolates belonged to different species or genera, e.g. *Fusarium* and *Cladosporium* (Table 2). Therefore, those results could not be used for reliable identification of fungi. Only the band obtained for *Metschnikowia pulcherrima* significantly differed from other tested microorganisms and could be useful for its initial identification. On the other hand, the band detected for *Metschnikowia* sp. isolate LW18 had the length similar to the majority of other fungi isolated in both years. Although the majority of tested microorganisms belonged to the *A. pullulans* species, their band sizes varied. On the other hand, in the case of RAPD-PCR, *A. pullulans* isolates collected in 2016 had one common band (1190 bp) while isolates obtained in 2017 were more variable (Table 2).

**Table 2:** The results of PCR carried out with ITS1 and ITS4 primers and RAPD-PCR obtained for fungal strains isolated in two subsequent harvest seasons

Year	Microorganism name	Isolate no.	PCR band size (bp)	RAPD-PCR band sizes (bp)
2016	*Candida railenensis*	LW24	623	3712, 3220, 1698, 1108
	*Aureobasidium pullulans*	LW25	610	3071, **1190**
	*Pichia membranifaciens*	LW26	613	1658
	*Aureobasidium pullulans*	LW12	595	**1190**
	*Aureobasidium pullulans*	LW10	595	3071, **1190**
	*Metschnikowia pulcherrima*	LW8	379	–
	*Aureobasidium pullulans*	LW80	582	3071, **1190**
	*Alternaria alternata*	LW76	574	–
	*Alternaria* sp.	LW77	560	598, 531
2017	*Aureobasidium pullulans*	LW81	576	3070, 2758, **1211**
	*Aureobasidium pullulans*	LW13	576	–
	*Aureobasidium pullulans*	LW14	566	3671, **1211**
	*Rhodotorula mucilaginosa*	LW15	605	3070, 2858, 2661, 2306, 1999
	*Aureobasidium pullulans*	LW16	570	3070, 2858, 2072, 1169
	*Aureobasidium pullulans*	LW17	551	**1211**
	*Metschnikowia* sp.	LW18	556	–
	*Aureobasidium pullulans*	LW19	564	3182, **1211**
	*Aureobasidium pullulans*	LW20	564	**1211**
	*Aureobasidium pullulans*	LW21	564	1169
	*Aureobasidium pullulans*	LW22	564	3070, 1128
	*Fusarium* sp.	LW23	539	614
	*Fusarium* sp.	LW78	525	400
	*Cladosporium* sp.	LW79	539	2567, 1999

Band sizes marked in bold in each year indicate bands which were common for most *A. pullulans* isolates obtained in each season

### Phylogenetic analysis and structural analysis of obtained sequences

**Fig. 1:**
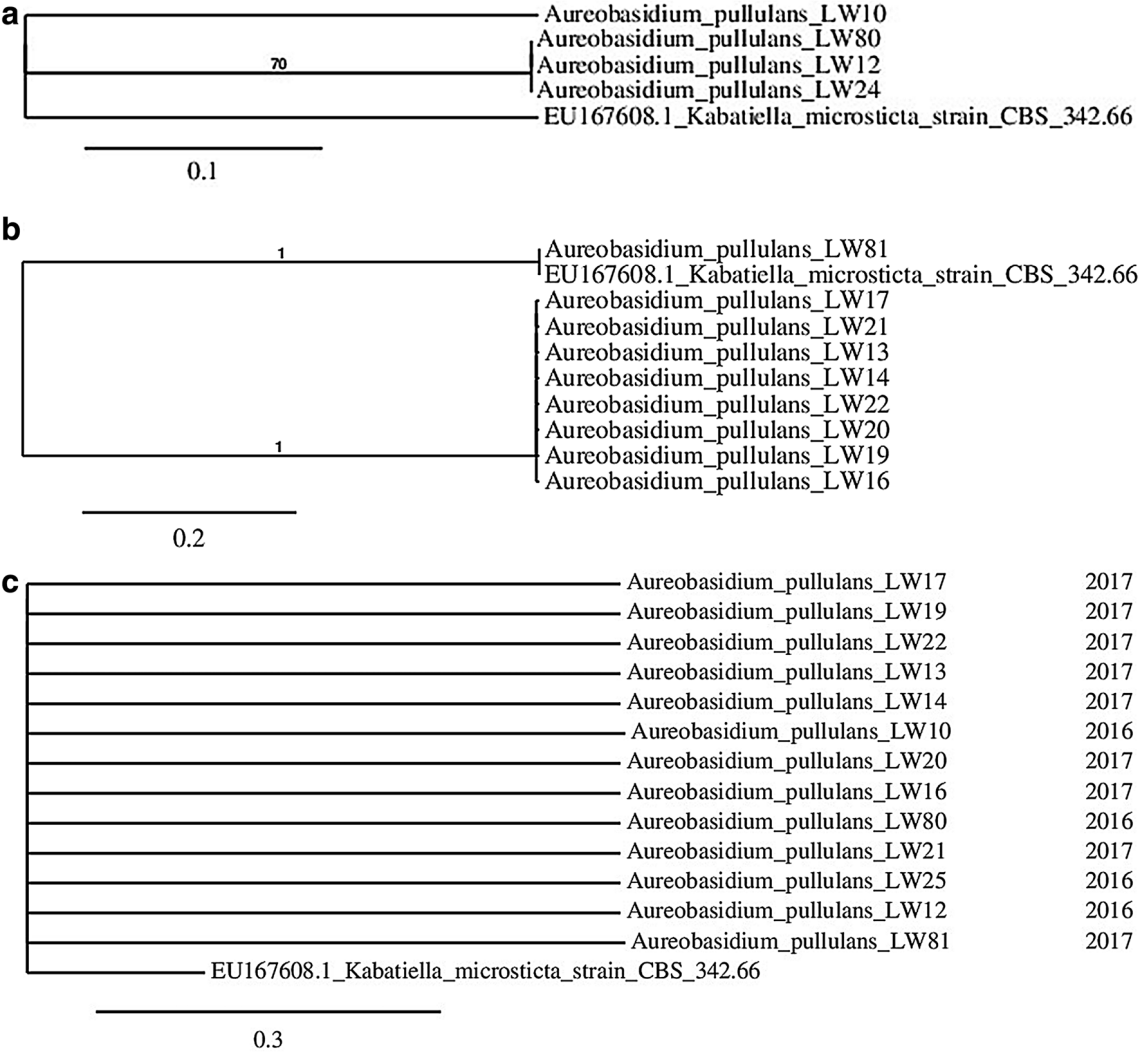
Phylogenetic tree obtained for *A. pullulans* isolates collected in 2016 (**a**), in 2017 (**b**) and in both years (**c**)

**Fig. 2:**
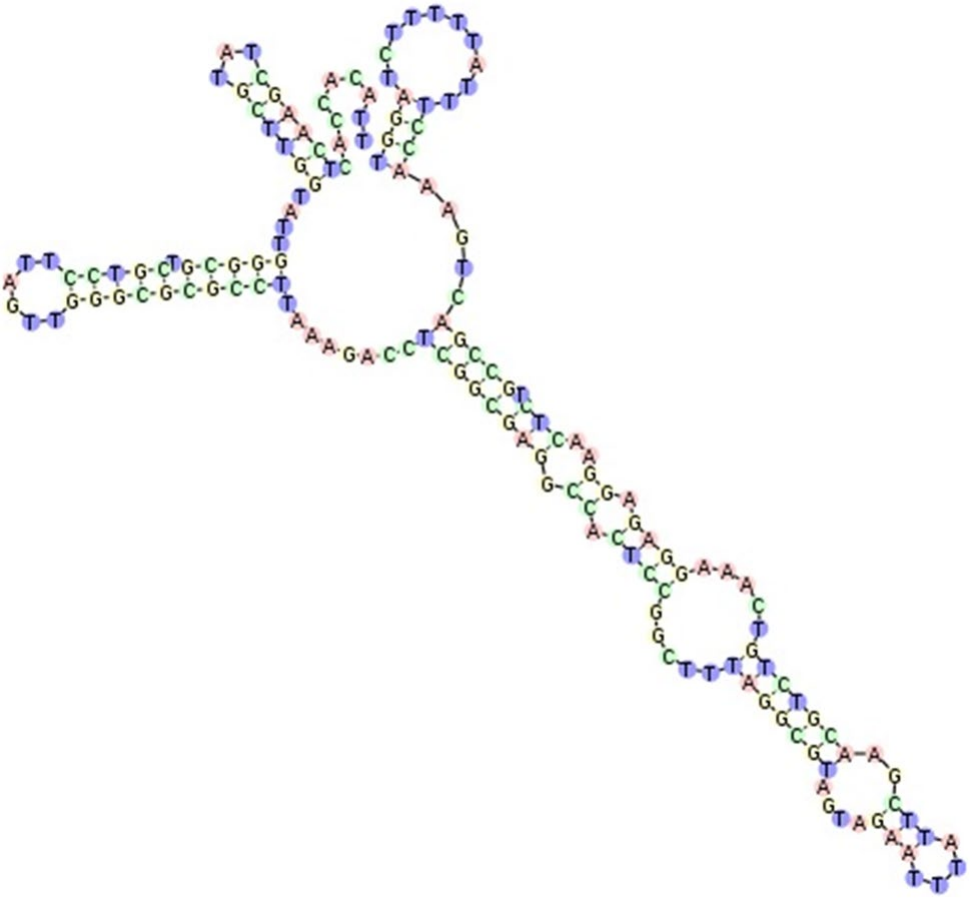
Secondary ITS2 region structure for *A. pullulans* isolate LW81. The structure was homology modelled by *Aureobasidium* sp. JSKim-2015 (accession number: LC018758.1, GI number: 747743838). The score calculated for that model was 675, detected gaps 3/163 (1.8%), identity 160/163 (98.2%) and similarity 160/163 (98.2%). Helix transfer was as follows: Helix 1: 100%, Helix 2: 100%, Helix 3: 100%, Helix 4: 100%

Phylogenetic analysis carried out for *A. pullulans* isolates collected in both years of the study, indicated strong divergence because most of branches had to be dropped since their support values were < 70% (Fig. 1a). Even when sequences were considered separately for particular year (Fig. 1b, c), these did not tend to form common clusters. Branch lengths showed significant number of nucleotide substitutions per site. Especially isolate LW81 differed from other tested microorganisms. Those differences were explained when we carried out the analysis of LW81 sequence in the ITS2 Database—it was the only sequence which we managed to annotate with 5.8S and 28S motifs and as a result ITS2 region was found and then its secondary structure was predicted (Fig. 2). In all other cases, it was not possible to obtain the sequence of ITS2 region.

**Fig. 3:**
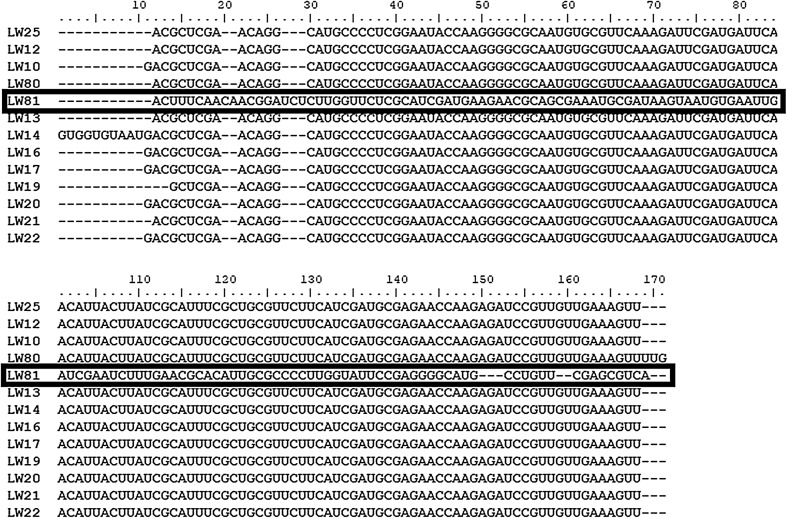
The alignment of 5.8S gene sequences of *A. pullulans* isolates obtained in Rfam. The alignment was based on MUSCLE algorithm. The sequence marked in bold indicates isolate which differed significantly from all other analysed microorganisms

To annotate 5.8S gene sequences in all analysed structures, we transcribed DNA to RNA and examined converted sequences in Rfam database. In the case of LW81 isolate the database allocated partial sequence of 28S gene as well (Table 3). 5.8S gene is highly conservative region in ribosomal DNA of fungi so after defining it in each analysed isolate we made an alignment in phylogeny.fr based on MUSCLE algorithm. It also confirmed that the sequence of the isolate LW81 varied from all others while other sequences demonstrated high homology (Fig. 3). Phylogenetic tree obtained with 5.8S sequences also did not indicate homology of obtained isolates and support values of branches were not satisfactory (below 70%, data not shown).

**Table 3:** Location of ITS1 and ITS2 regions and 5.8S and partial 28S genes in *A. pullulans* sequences

Year	Isolate no.	ITS1	5.8S	ITS2	28S
2016	LW25	1–140	141–294	295–450	451–500
	LW12	1–147	148–301	302–457	458–500
	LW10	1–141	142–295	296–451	452–500
	LW80	1–141	159–315	316–461	462–510
2017	**LW81**	**1**–**136**	**137**–**290**	**291**–**450**	**451**–**510**
	LW13	1–158	159–312	316–461	462–521
	LW14	1–153	154–317	318–463	464–528
	LW16	1–158	159–312	313–457	458–521
	LW17	1–145	146–299	300–455	456–486
	LW19	1–140	141–294	295–450	451–501
	LW20	1–159	160–313	314–469	470–521
	LW21	1–161	162–315	316–471	472–521
	LW22	1–161	162–315	316–471	472–521
*A. pullulans* (KT693733.1)	CBS 584.75	12–191	192–348	349–504	505–542

In the case of isolate marked in bold ITS2 region was annotated in ITS2 Database

The next step in the current study was to analyse sequences in the BLASTn engine restricting the search only to culture type materials. The highest maximum score was obtained for *A. pullulans* strain CBS 584.75 (accession no. KT693733.1). That strain has got defined location of ribosomal DNA components so sequences considered in the current study were compared to those fragments to assess their locations in analysed sequences (Table 3).

### Identification of exopolysaccharide producers

**Fig. 4:**
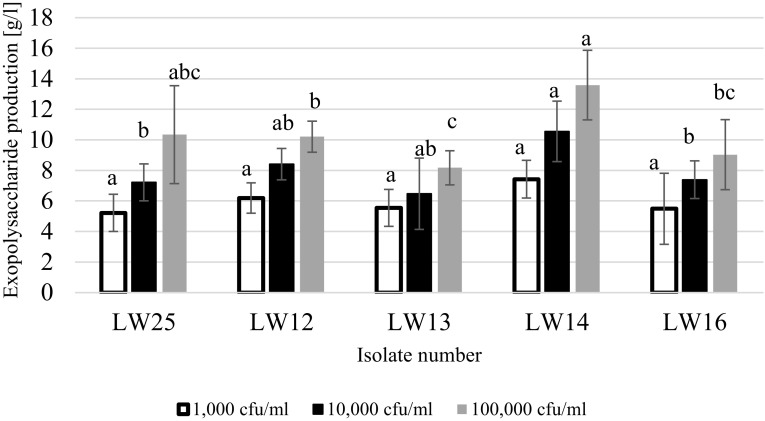
Exopolysaccharide production by selected isolates of *A. pullulans* at various initial cell numbers. The same letters above columns for each initial cell number indicate the lack of statistically significant differences at p < 0.05, n = 5

Only few strains of *A. pullulans* formed slimy colonies on Czapek-Dox medium, however, most of them managed to grow on that medium (Table 4). The majority of isolates did not produce black pigment. We selected five isolates for assessing their ability for forming exopolysaccharides in liquid medium: LW25, LW12, LW13, LW14 and LW16. It was demonstrated that the greatest quantities of exopolysaccharide were formed when initial cell number was 100,000 cfu/ml (Fig. 4) in all tested cases. The most potent exopolysaccharide producer was isolate LW 14 while LW13 demonstrated significantly weaker abilities to synthetase those compounds. Based on these findings, isolate LW14 was added to the apple juice and subjected to pasteurisation. The results indicated that selected isolate did not survive pasteurisation.

**Table 4:** Screening for exopolysaccharide producers among *A. pullulans* strains obtained in two subsequent harvest seasons

Year	Isolate no.	Colony appearance	Growth intensity	Intensity of exopolysaccharide production
2016	LW25	Black-brown colonies, small round colonies	++++	++++
	LW12	Black-brown colonies, small round colonies	++++	++
	LW10	Black-brown colonies with wrinkled surface	+++	–
	LW80	No growth	–	–
2017	LW81	Black-brown colonies with wrinkled surface, small round colonies	++++	–
	LW13	Black-brown colonies, small round colonies	++++	++++
	**LW14**	**Black-brown colonies, small round colonies**	+++++	++++++
	LW16	Brown colonies with wrinkled surface	+++	++
	LW17	Beige-brown colonies	+++	–
	LW19	Brown colonies	++	–
	LW20	No growth	–	–
	LW21	Beige colonies, small round colonies	++++	–
	LW22	Black colonies with wrinkled surface	++++	–

‘–’ No growth/slimy colonies formation; ‘+’ very weak growth/slight colony sliminess; ‘++’ weak growth/slight colony sliminess; ‘+++’ moderate growth/slight colony sliminess; ‘++++’ good growth/slight colony sliminess; ‘+++++’ very good growth/slight colony sliminess. Isolate marked in bold was the most significant exopolysaccharide producer

## Discussion

According to our knowledge, our study was the first to demonstrate that *C. railenensis* might be present in apples—it was previously found on the fruit of an English oak (Isaeva et al. 2009) or on the decayed wood in the evergreen forest (Ramírez and González 1984). Its presence in apple fruit is not surprising since there are oak trees growing in the neighbourhood of the orchard where fruits for the current research were harvested. The presence of *A. pullulans* in apple orchards has been already reported (Bernier et al. 1996; Mirzwa-Mróz et al. 2014). However, studies carried out by Bernier et al. (1996) were conducted without application of molecular biology methods so the results might have been biased by the inaccuracy of classical methods used for microbial identification. In the study conducted by Mirzwa-Mróz et al. (2014) PCR product of only one isolate (out of 16) was sequenced so there is still very little known about *A. pullulans* isolates collected from apples. Moreover, we demonstrated that band sizes obtained after amplification with IST1 and ITS4 primers do not provide conclusive information for the identification of microorganisms. Additionally, results of RAPD-PCR with M13 can not be used as a reference in further research since that method was proven inefficient and unreliable (Gil-Lamaignere et al. 2003). On the other, application of that method could be useful for differentiation of obtained isolates. To make that method feasible for *A. pullulans*, other primers need to be selected. Based on our experience, RAPD-PCR needs to be carried out with at least two different primers. In our case all isolates demonstrated various band patterns, therefore, it was not necessary to search for another primer.

Surprisingly, we did not detect any *Penicillium* isolates, despite the fact they commonly reside in apple in various geographic regions (Bernier et al. 1996; Scheper et al. 2007; Welke et al. 2010; Alwakeel 2013). Their absence could be related to the abundance of *A. pullulans* which is considered as a biocontrol agent effectively inhibiting the growth of *Penicillium* species (Mari et al. 2012). We also identified one isolate of *P. membranifaciens* which could be potentially a producer of killer toxins (Muccilli and Restuccia 2015) and these compounds are known to inhibit fungal growth as well. Moreover, the current study was not focused on the quantitative analysis of each fungal species so there is a chance that there were *Penicillium* strains among isolates which were not selected for DNA extraction. Other mould found in fruit in the current study—*Fusarium, Alternaria* and *Cladosporium*, commonly reside in apple (Bernier et al. 1996; Grantina-Ievina 2015; Abdelfattah et al. 2016). In the study carried out in Italy, also the presence of *M. pulcherrima* and some *Pichia* and *Rhodotorula* species has been confirmed (Pelliccia et al. 2011).

Isolates of *A. pullulans* obtained in the current study demonstrated great divergence which was transparent in their growth characteristics on WL nutrient agar and Czapek-Dox agar but also in their ability to produce exopolysaccharides. These observations were confirmed by the results of phylogenetic analysis: the alignment which was prepared in the first stage clearly indicated that sequences were not homologous so there were no two the same isolates that resided in both years of the study. There are various scenarios to explain this phenomenon. Nowadays, especially small companies which have their own orchards, purchase cultivars of various fruit from small local farms and then deliver goods to supermarkets or shops in cities. Due to the annual changes of weather, amount of precipitation, average seasonal temperatures and ground frost, the selection of local farms might vary from 1 year to another because all mentioned factors would influence the yield of fruit. Therefore, each year microorganisms are transferred from one orchard to another, from the area that might sometimes reach up to 20 km (personal hearing). Another possible route for transmitting microorganisms is planting young trees to replace old ones. Also, due to changes in customer demands, it is necessary to implement novel fruit varieties or to import varieties which are not common for the region where orchard is located.

Another important aspect which was revealed in the phylogenetic analysis and extracting DNA regions is that the gene pool within *A. pullulans* species could be changing. There was only one isolate (LW81) which resembled already known sequences stored in the GenBank while all others (12 isolates) represented new cluster, perhaps a novel variety of *A. pullulans*. Changes in the genome could be a response to various factors, including implementation of innovations to horticultural practices, e.g. new types of fungicide or fertiliser. On the other hand, there is very little data available on microorganisms obtained from apple so before making ambiguous conclusions, more data should be collected and analysed.

In the industrial production of pullulan, more than 70% yields of the initial carbon sources are obtained (Leathers 2003). In the studies focused on optimisation of pullulan production, 29 g/l was obtained after 84 h fermentation (Yoon et al. 2012) while it was 17.2 g/l when synthetic medium was used (Göksungur et al. 2005). In the current study, isolate LW14 produced 13.58 g of exopolysaccharide/l. These differences could be caused by various concentrations of sucrose, which was 165.73 g/l (Yoon et al. 2012), 51.4 g/l (Göksungur et al. 2005) and 30 g/l (current study), respectively. Other factors that could contribute to these outcomes were: fermentation time, various concentrations of mineral salts and aeration rates. Since optimisation of exopolysaccharide production was not the main focus of the current study, no further experiments were carried out in that direction. On the other hand, isolate LW14 seems to be very promising candidate for pullulan production so in the future studies should be continued to optimise exopolysaccharide production.

It was demonstrated that the isolate LW14 should not pose any risk to the quality of pasteurised apple juice. Further studies should be initiated to verify how this particular isolate affects the quality of unpasteurised juices. It is a group of products which is gaining a lot of consumer interest.

## References

[CR1] Abdelfattah A, Wisniewski M, Droby S, Schena L (2016). Spatial and compositional variation in the fungal communities of organic and conventionally grown apple fruit at the consumer point-of-purchase. Hortic Res.

[CR2] Alwakeel SS (2013). Molecular identification of isolated fungi from stored apples in Riyadh, Saudi Arabia. Saudi J Biol Sci.

[CR3] Andrighetto C, Psomas E, Tzanetakis N (2000). Randomly amplified polymorphic DNA (RAPD) PCR for the identification of yeasts isolated from dairy products. Lett Appl Microbiol.

[CR4] Aneja KR, Dhiman R, Aggarwal NK, Aneja A (2014). Emerging preservation techniques for controlling spoilage and pathogenic microorganisms in fruit juices. Int J Microbiol.

[CR5] Bernier J, Carisse O, Paulitz TC (1996). Fungal communities isolated from dead apple leaves from orchards in Québec. Phytoprotection.

[CR6] Castresana J (2018). Selection of conserved blocks from multiple alignments for their use in phylogenetic analysis. Mol Biol Evol.

[CR7] Cheng K-C, Demirci A, Catchmark JM (2011). Pullulan: biosynthesis, production, and applications. Appl Microbiol Biotechnol.

[CR8] Chevenet F, Brun C, Bañuls AL (2006). TreeDyn: towards dynamic graphics and annotations for analyses of trees. BMC Bioinform.

[CR9] Dereeper A, Guignon V, Blanc G (2008). Phylogeny.fr: robust phylogenetic analysis for the non-specialist. Nucleic Acids Res.

[CR10] Edgar RC (2004). MUSCLE: Multiple sequence alignment with high accuracy and high throughput. Nucleic Acids Res.

[CR11] Fujita S-I, Senda Y, Nakaguchi S, Hashimoto T (2001). Multiplex PCR using internal transcribed spacer 1 and 2 regions for rapid detection and identification of yeast strains. J Clin Microbiol.

[CR12] Gil-Lamaignere C, Roilides E, Hacker J, Müller FMC (2003). Molecular typing for fungi—a critical review of the possibilities and limitations of currently and future methods. Clin Microbiol Infect.

[CR13] Göksungur Y, Dǎgbaǧli S, Uçan A, Güvenç U (2005). Optimization of pullulan production from synthetic medium by *Aureobasidium pullulans* in a stirred tank reactor by response surface methodology. J Chem Technol Biotechnol.

[CR14] Gostinčar C, Ohm R, Kogej T (2014). Genome sequencing of four *Aureobasidium pullulans* varieties: biotechnological potential, stress tolerance, and description of new species. BMC Genomics.

[CR15] Grantina-Ievina L (2015). Fungi causing storage rot of apple fruit in integrated pest management system and their sensitivity to fungicides. Rural Sustain Res.

[CR16] Guindon S, Dufayard JF, Lefort V (2010). New algorithms and methods to estimate maximum-likelihood phylogenies: assessing the performance of PhyML 3.0. Syst Biol.

[CR17] Hall TA (1999). BioEdit: a user-friendly biological sequence alignment editor and analysis program for Windows 95/98/NT. Nucleic Acids Symp Ser.

[CR18] Isaeva OV, Glushakova AM, Yurkov AM, Chernov IY (2009). The yeast *Candida railenensis* in the fruits of English oak (*Quercus robur* L.). Microbiology.

[CR19] Kalvari I, Argasinska J, Quinones-Olvera N (2018). Rfam 13.0: shifting to a genome-centric resource for non-coding RNA families. Nucleic Acids Res.

[CR20] Koetschan C, Förster F, Keller A (2009). The ITS2 Database III—sequences and structures for phylogeny. Nucleic Acids Res.

[CR21] Leathers TD (2003). Biotechnological production and applications of pullulan. Appl Microbiol Biotechnol.

[CR22] Mari M, Martini C, Spadoni A (2012). Biocontrol of apple postharvest decay by *Aureobasidium pullulans*. Postharvest Biol Technol.

[CR23] Merget B, Koetschan C, Hackl T (2012). The ITS2 Database. J Vis Exp.

[CR24] Mirzwa-Mróz E, Dzięcioł R, Pitera E, Jurkowski A (2012). Influence of sooty blotch and flyspeck (SBFS) fungi on apple fruits during storage. Acta Sci Pol Hortorum Cultus.

[CR25] Mirzwa-Mróz E, Wińska-Krysiak M, Dzięcioł R, Miękus A (2014). Characteristics of *Aureobasidium pullulans* (de Bary et Löwenthal) G. Arnaud isolated from apples and pears with symptoms of sooty blotch in Poland. Acta Sci Pol Hortorum Cultus.

[CR26] Muccilli S, Restuccia C (2015). Bioprotective role of yeasts. Microorganisms.

[CR27] Nawrocki EP, Burge SW, Bateman A (2015). Rfam 12.0: updates to the RNA families database. Nucleic Acids Res.

[CR28] Pelliccia C, Antonielli L, Corte L (2011). Preliminary prospection of the yeast biodiversity on apple and pear surfaces from Northern Italy orchards. Ann Microbiol.

[CR29] Pennycook SR, Newhook FJ (1981). Seasonal changes in the apple phylloplane microflora. N Z J Bot.

[CR30] Ramírez C, González A (1984). Five new filamentous, glucose-fermenting *Candida* isolated from decayed wood in the evergreen rainy Valdivian forest of southern Chile. Mycopathologia.

[CR31] Scheper RWA, Rogers DJ, Walker JTS (2007). The incidence of storage rots after postharvest apple washing. N Z Plant Prot.

[CR32] Street G, Kingdom U (2006). Approximate likelihood-ratio test for branches: a fast, accurate, and powerful alternative. Syst Biol.

[CR33] Wareing P, Davenport RR (2007). Microbiology of soft drinks and fruit juices. Chemistry and technology of soft drinks and fruit juices.

[CR34] Welke JE, Hoeltz M, Dottori HA, Noll IB (2010). Fungi and patulin in apples and the role of processing on patulin levels in juices: a study on naturally contaminated apples. J Food Saf.

[CR35] Williams AJ (1955) Changes in the microflora of apples during ripening and cold storage. McGill University

[CR36] Yoon S, Hong E, Kim S (2012). Optimization of culture medium for enhanced production of exopolysaccharide from *Aureobasidium pullulans*. Bioprocess Biosyst Eng.

[CR37] Zalar P, Gostinčar C, de Hoog GS (2008). Redefinition of *Aureobasidium pullulans* and its varieties. Stud Mycol.

